# Topical Administration of Drugs Incorporated in Carriers Containing Phospholipid Soft Vesicles for the Treatment of Skin Medical Conditions

**DOI:** 10.3390/pharmaceutics13122129

**Published:** 2021-12-10

**Authors:** Elka Touitou, Hiba Natsheh

**Affiliations:** The Institute for Drug Research, School of Pharmacy, Faculty of Medicine, The Hebrew University of Jerusalem, Ein Kerem, P.O. Box 12065, Jerusalem 9112102, Israel; hiba.natsheh@mail.huji.ac.il

**Keywords:** soft phospholipid vesicle, skin disorders, ethosomes, glycerosomes, transethosomes, skin infection, skin inflammation, skin cancer, acne vulgaris, wound healing, hair loss, psoriasis, melasma, vitiligo

## Abstract

This review focuses on the improved topical treatment of various medical skin conditions by the use of drugs delivered from carriers containing phospholipid soft vesicles. Topical drug delivery has many advantages over other ways of administration, having increased patient compliance, avoiding the first-pass effect following oral drug administration or not requesting multiple doses administration. However, the skin barrier prevents the access of the applied drug, affecting its therapeutic activity. Carriers containing phospholipid soft vesicles are a new approach to enhance drug delivery into the skin and to improve the treatment outcome. These vesicles contain molecules that have the property to fluidize the phospholipid bilayers generating the soft vesicle and allowing it to penetrate into the deep skin layers. Ethosomes, glycerosomes and transethosomes are soft vesicles containing ethanol, glycerol or a mixture of ethanol and a surfactant, respectively. We review a large number of publications on the research carried out in vitro, in vivo in animal models and in humans in clinical studies, with compositions containing various active molecules for treatment of skin medical conditions including skin infections, skin inflammation, psoriasis, skin cancer, acne vulgaris, hair loss, psoriasis and skin aging.

## 1. Introduction

The use of drug delivery systems for the treatment of skin medical conditions was introduced long time ago. Extensive research has been carried out to improve the local treatment of various skin diseases such are wounds, psoriasis, microbial infections, acne, inflammation, autoimmune diseases and skin cancer.

Topical drug administration via the skin route has many advantages including targeting the place of action, a decreased need for multiple doses and a high patient compliance [1]. However, in cases in which the disease does not affect the stratum corneum (SC), most molecules are unable to efficiently cross this skin barrier.

The skin is the largest organ accounting for about 15% of the total adult body weight and covering the whole human body surface. The SC is the outermost layer of the skin, and consists of nonviable, anucleate, keratinized cells. This layer forms an effective barrier to retain water within the body and to prevent the access of exogenous compounds. This explains the reported lack of efficiency of many drugs following their administration in conventional carriers. For example, many topical creams and ointments of the antiviral agent, acyclovir, have a minor clinical effectiveness [2]. The properties of the SC are mainly related to its content of lipids, ceramides, cholesterol and fatty acids. These lipids are packed into small organelles forming lamellar granules organized in a fused edge-to-edge manner which appear microscopically as flattened lamellar disks ordered in paired bilayers [3]. The main penetration pathway for molecules is though this layer via the intracellular route [4]. The water concentration in this layer is around 25% only. The viable epidermis and the dermis, the skin deeper layers represent the hydrophilic region containing a high-water concentration.

In order to improve the access of active molecules into the skin, penetration enhancers have been designed [5]. These are molecules that mainly interact with SC lipid bilayers to promote the drug flux into this tissue. The most investigated penetration enhancers are fatty acids, solvents, DMSO and derivatives, urea, surfactants, polysaccharides, and pyrrolidones [6,7,8]. In the last four decades, nanocarriers were designed for drug dermal delivery used in the treatment of various medical conditions. The nanocarriers include solid lipid nanoparticles, nanovesicular carriers, nanospheres, microemulsions, nanoemulsions, and polymeric nanoparticles [5,9].

**Figure 1:**
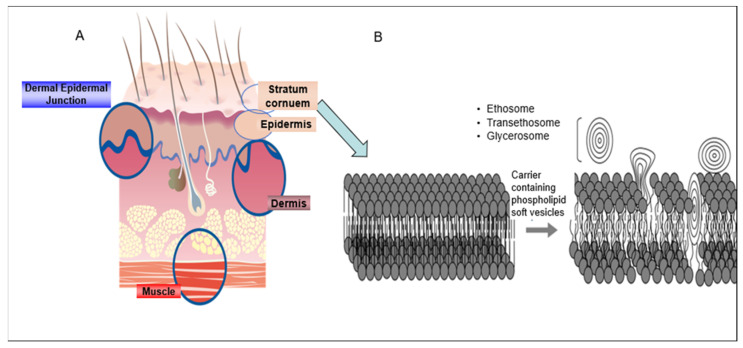
Schematic illustration of (**A**) the skin structure (**B**) the mechanism of action of carriers containing phospholipid soft vesicles.

About four decades ago, new skin penetration enhancing delivery carriers containing phospholipid soft vesicles were designed. These phospholipid vesicles are characterized by their fluid lipid bilayers owing to the presence of molecules such as ethanol and glycols. Upon topical administration, the solvents in these carriers fluidize and disrupt the bilayers of the lipids in the SC and facilitate the penetration of the soft vesicles deeper into the skin, where their active content is released [10,11,12,13] (Figure 1). An increasing attention has been given for the treatment of skin medical conditions via topical administration of drugs incorporated in these new carriers.

The ethosome is the first and most investigated soft vesicle. These delivery systems containing the soft vesicles were introduced by Touitou in 1998 [11]. Drug ethosomal systems are composed of phospholipid, ethanol (20–50% *w*/*w*) with, or without glycols, water and an active molecule. In general, ethosomes are multilamellar nanovesicles containing bilayers from wall to wall. The high alcohol content in ethosomal systems fluidizes the phospholipid bilayers producing soft vesicles with significant decrease in the transition temperature (Tm) as measured by differential scanning calorimetry (DSC) compared with conventional liposomes. For example, a decrease of 21.5 °C in the Tm of the phospholipid vesicles was measured for ethosomes not containing an active molecule and those containing bacitracin or erythromycin as compared to the corresponding liposome. In addition, ethosomal systems containing ibuprofen had a Tm lower by 24.6 °C as compared to liposomes containing equal concentration of the drug [10,11,14,15]. Small and large active lipophilic and hydrophilic molecules were successfully incorporated in ethosomes. These systems were investigated for the treatment of a wide variety of skin diseases such as bacterial, fungal and viral infections, inflammation, atopic dermatitis, psoriasis, skin cancer and skin pigmentation disorders.

The next generation of soft vesicles were the transethosomes [12,16]. These are phospholipid carriers having the composition of ethosomes with the addition of surfactants such as Tween 80, sodium taurocholate, oleic acid or decyl methyl sulfoxide [12,16].

Glycerosomes, other systems containing soft vesicles, were first described by Manca et al. [13]. These carriers contain glycerol, at a concentration range of 10–30% *v*/*v*. The authors reported a decrease of 0.5–0.9 °C in the Tm of the vesicles compared to the corresponding liposomes. Interestingly, the effect of glycerol on the vesicles fluidity is minor compared to that of ethanol in ethosomes [17].

Medical applications of ethosomes in the field of dermal drug administration have been extensively reviewed [18,19,20,21,22,23,24].

Delivery systems containing phospholipid soft nanovesicles are biocompatible, biodegradable and have a low toxicity profile. In addition, these carriers can improve the delivery of poorly water-soluble drugs through several administration routes [25]. They have been designed to overcome the limited ability of the classic phospholipid vesicles, such liposomes, to facilitate drug penetration into the deep skin layers [10,26].

In a previous review, we focused on the characteristics of the various types of carriers containing altered phospholipid vesicles. The effect of agents such as ethanol and surfactants on the structure of each type of vesicle, its mechanism of action and application mode on the skin were comprehensively described [17].

In the present analysis we are emphasizing the contribution of drug delivery systems containing phospholipid soft vesicles to the efficiency of treatment of various skin medical conditions.

## 2. Treatment Approaches with Drugs Incorporated in Carriers Containing Phospholipid Soft Vesicles

**Figure 2:**
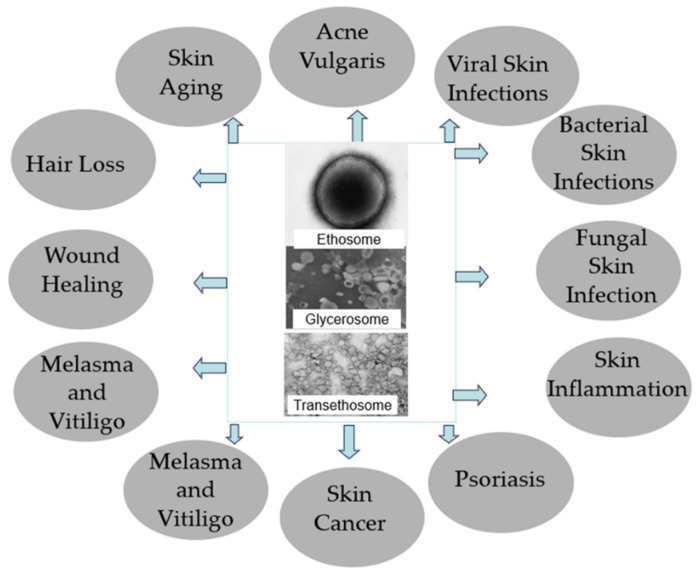
Skin disorders treated with topical drug delivery systems containing phospholipid soft vesicles.

Drug delivery systems containing phospholipid soft vesicles have been widely investigated for the treatment of skin disorders including skin infections, skin inflammation, acne vulgaris, skin pigmentation disorders, hair loss and skin cancer (Figure 2). This section covers in vitro, preclinical and clinical studies conducted to evaluate the ability of these carriers to improve the treatment outcome.

### 2.1. Treatment of Acne Vulgaris

Acne vulgaris is one of the commonest skin disorders which mainly affect adolescents. Clinical symptoms of this condition include comedones, seborrhoea, erythematous papules and pustules. Nodules, deep pustules, and ultimate scarring are symptoms of severe acne. The pathogenesis of acne involves four mechanisms: follicular hyperkeratinization, *Propionibacterium acne* (*P. acne*) colonization, increased sebum production and local inflammation [27,28,29].

Owing to a better understanding of the pathogenesis of acne, new therapeutic modalities have been designed. Treatment of acne vulgaris is based of systemic or topical administration of therapy agents. Retinoids, antibiotics, benzoyl peroxide, salicylic acid and azelaic acid are the most prescribed topical medications for acne treatment [30]. These compounds are conventionally administrated in solution, gel or lotion-based formulations.

**Figure 3:**
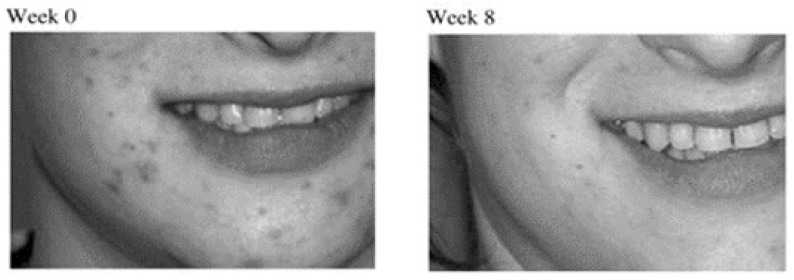
Photographs of a patient with moderate acne treated with clindamycin phosphate and salicylic acid ethosomal gel for 8 weeks at the baseline (week 0) and at the end of the study (week 8) (Reprinted with permission from ref. [31]. Copyright 2008 Wiley).

Touitou et al. [31] investigated an ethosomal gel containing clindamycin and salicylic acid for the treatment of acne vulgaris in a randomized double-blind clinical study of 8-weeks on 40 patients. The results indicated that in patients with mild to moderate symptoms, a significant reduction in the clinical signs including the number of inflammatory, non-inflammatory and total lesions was observed following the 8-weeks treatment (Figure 3). More than 70% of the participants reported partial or complete improvement. In addition, more than 80% of the patients, who underwent previous treatment with 1% clindamycin lotion, 5–10% benzoyl peroxide gels, 5% benzoyl peroxide–2% erythromycin gel, reported a better tolerability of the ethosomal gel with less burning, pruritus, erythema, and photosensitivity reactions.

This superior effect of the ethosomal system is mainly attributed by the ethanol high concentration which fluidizes the bilayers of the phospholipid vesicles and the lipid membranes in the SC of the treated skin. Upon topical administration of ethosomes, a dual mechanism of penetration enhancement takes place; the soft ethosome with fluid bilayer penetrates easily through the disrupted SC bilayers and reaches deeper into the skin where releases its drug content [10].

Another work describing the use of azelaic acid ethosomal systems for the treatment of acne vulgaris was published by Esposito et al. [32]. Ethosomal and liposomal dispersions of azelaic acid incorporated in Carbopol© 934P gel were designed and investigated. The active ingredient used in this work is a saturated natural dicarboxylic acid with activity against microbial colonization in the pilosebaceous unit in the skin, inflammation of the perifollicular area, sebum production and excretion and keratinization of the follicular channel. Diffusion studies through synthetic membranes indicated a rapid release rate of azelaic acid from the ethosomal systems containing higher ethanol concentration. For example, ethosomal dispersions containing 20 and 40% ethanol yielded release rates of 87.78 and 119.96 µg·cm^−2^·min^−0.5^, respectively. These values were up to 2-times higher than those achieved with the liposomal dispersion which yielded a release rate of 59.63 µg·cm^−2^·min^−0.5^. The authors also reported that the gel systems exhibited slower release rates yet maintained a higher release from ethosome versus liposome.

Yu et al. [33] designed ethosomes loaded with the natural anti-inflammatory molecule, cryptotanshinone (CPT) and incorporated them in a Carbomer 974 gel. The formulations were investigated for acne management as compared to conventional hydroethanolic gels. The anti-acne activity and skin irritation of the gel were evaluated in the oleic acid-induced acne rabbit model. Following treatment with CPT loaded ethosomes, the affected skin recovered and returned to its normal structure. No evidence of inflammation in the pilosebaceous unit or keratoplasia in the follicles and SC were observed. Lymphatic cell infiltration in the dermis was not observed. The epidermis had a similar thickness to that of the normal skin. This was in contrary to the outcomes of conventional gel treatment where keratoplasia and inflammation were present in the pilosebaceous follicles and SC.

Ansari et al. [34] incorporated the anti-inflammatory molecule karanjin in an ethosomal gel to enhance its topical delivery and activity against acne. The in vitro experiment using rat skin and Franz diffusion cells indicated a 2.4 higher deposition of the molecules in the animal skin than that of the solution. In addition, their formulation was found to be non-irritant to the skin as shown by Draize score which tested erythema, edema and scar formation. The anti-acne effect was also evident with the ethosomal treatment with a significant decrease in the number and size of the sebaceous gland units in dermis, as shown by the histopathologic examination. Furthermore, substantial anti-inflammatory effects in the carrageenan-induced edema in the rat paw were evident with inhibition of rat paw edema by 66.66 and 70.37% upon application of the ethosomal system and a clindamycin containing marketed formulation (0.5% gel), respectively.

The studies on acne vulgaris and other medical applications for carriers containing drugs incorporated in phospholipid soft vesicles are presented in Table 1.

### 2.2. Treatment of Viral Skin Infections

A significant percentage of skin diseases are caused by viral infections. Clinical manifestation of these disorders mainly include skin rash and lesions in the mucosal membrane, pain and discomfort. Herpes simplex (HSV) is the commonest cause of these disorders. The virus belongs to the herpes viridae family and has 2 types; type 1 and type 2. HSV-1, also known as herpes labialis, is the primary cause of cold sores, while HSV-2 leads to genital herpes [72]. Topical acyclovir is the first-line treatment of HSV infections. Other treatments include topical pencyclovir and orally administrated valacyclovir and famciclovir. Acyclovir selectively inhibits viral DNA polymerase, preventing further elongation of the nucleic acid chain and stopping the virus replication. Since the introduction of this antiviral agent, nearly 2 decades ago, its topical administration has been extensively investigated for the treatment of Herpes simplex. Unlike oral acyclovir treatments, topical therapy allows administration of smaller amounts of the drug, which is applied directly to the infected target site. Using this route, the dug systemic adverse effects can be reduced. Ointments and creams containing acyclovir at a concentration of 5%, applied every 3 h are the conventional topical dosage forms [2]. However, some clinical studies indicated that these products are of minor clinical effectiveness [73,74,75,76]. This could be due to non-sufficient penetration of the molecule into the basal epidermis, where the virus replicates. To answer the need of an improved treatment, acyclovir has been formulated using skin penetration enhancing carriers containing soft vesicles.

**Figure 4:**
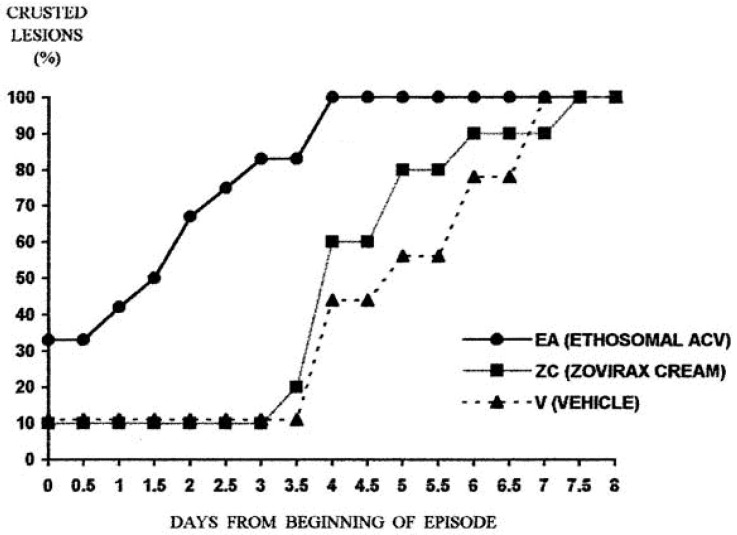
Days to crust formation: parallel arm. Day 0: 33% of lesions aborted in EA subgroup, 10% in ZC subgroup, 11% in V subgroup. Day 3: 80% of lesions crusted in EA subgroup, 10% in ZC subgroup, 11% in V subgroup. Day 4: 100% of lesions crusted in EA subgroup, 60% in ZC subgroup, 44% in V subgroup. Time to crusting of all lesions: 4 days in EA subgroup, 7 days in ZC subgroup, 7.5 days in V subgroup (Reprinted with permission from ref. [35]. Copyright 1999 Elsevier).

The first clinical evaluation of such a system was published by Touitou’s group [35]. A double-blind, crossover 2-armed, randomized study was conducted on 40 subjects with recurrent herpes labialis. The study aimed to test the antiviral efficacy 5% acyclovir ethosomal system compared to the commercial cream (Zovirax^®^) and to ethosomal carrier not containing the drug. The reported results of the parallel part of the study indicated that the time for lesions crusting in the group treated with ethosomal system was 1.6 vs. 4.3 and 4.8 days for the commercial product and the empty vehicle, respectively. Lesions crusting time of 1.8 and 3.5 days for ethosomal system and the commercial cream, respectively, were obtained in the crossover section. Full crusts recovery was reported 4.2, 5.9 days after treatment with acyclovir ethosomal system and the Zovirax^®^ cream, respectively (Figure 4). Regarding other clinical parameters (such as the proportion of abortive lesions) an improvement was stated in 30% of episodes treated with ethosomes, compared to 10% of episodes treated with Zovirax.

**Figure 5:**
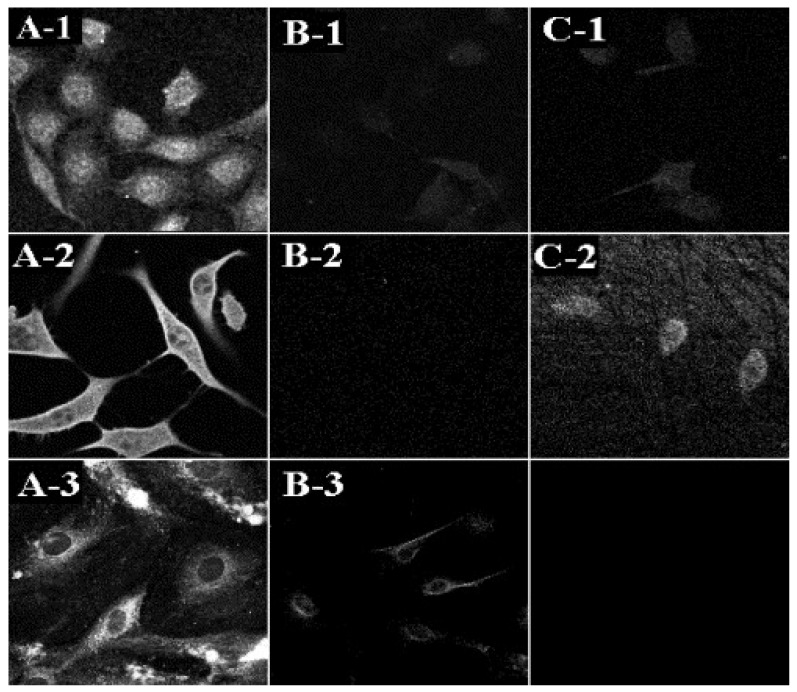
CLS micrographs showing intracellular fluorescence in fibroblasts following delivery of various fluorescent probes by ethosomes and control systems. Fluorescent probes (D289: **A1**–**C1**, RR: **A2**–**C2** or PC*: **A3**, **B3**) were applied to 3T3 fibroblasts for 10 min from various systems as follows: ethosomes: **A1**–**A3**; liposomes: **B1**–**B3**; hydroethanolic solution: **C1**, **C2** (Reprinted with permission from ref. [77]. Copyright 2001 Elsevier).

This improved antiviral effect of acyclovir when applied topically in ethosome can be explained by the ability of the carrier loaded with the drug to target the virus inside the cells. First, the ethosomal system containing a high ethanol concentration disrupts and fluidizes the cellular membrane which allows the soft vesicle to fuse and release its drug content inside the cell. This hypothesized mechanism of action was supported by the enhanced intracellular delivery of the amphipathic probe 4-(4-diethylamino) styryl-*N*-methylpyridinium iodide (D-289), the lipophilic probe rhodamine red (RR) and the fluorescent phosphatidylcholine (PC*) into fibroblasts, as examined in vitro by confocal laser scanning microscopy (CLSM). As an example, the measured RR fluorescence intensities by CLSM were 150, 40 and 20 arbitrary unit (A.U.) for the probe delivered in ethosomes, hydroethanolic solution and liposomes, respectively (Figure 5) [77].

Recently, glycerosomes, carriers containing soft phospholipid vesicles owing to the presence of glycerol, have been investigated for treatment of HSV-1. Vanti et al. [38] incorporated the essential oil of Melissa officinalis, in these carriers as a strategy to increase their stability and enhance their antiviral activity against HSV type 1. The results of the in vitro experiment on mammalian cells indicated that glycerosomes containing the oil were efficient in inhibiting HSV type 1 infection, without producing cytotoxic effects.

### 2.3. Treatment of Bacterial Skin Infections

Skin infections such as cellulitis, erysipelas and trauma- and wound-related infections are among the most frequent conditions demanding acute ambulatory care. *Staphylococcus aureus* is the major cause of such conditions. Inappropriate treatment may lead to severe complications and hospital admission. In some cases, these infections may spread to other body parts causing serious morbidity and mortality.

Topical administration of antibiotics from conventional topical dosage forms is associated with limited penetration into the deep skin layer leading to poor prognosis and insufficient treatment efficiency. Oral or parenteral antibiotics at high doses of macrolides, β-lactams or other antibacterial agents are usually used to control such cases. However, systemic antibiotic treatment may increase the incidence of side effects and allergic reactions which may affect the patient convenience [78,79,80,81].

Topical administration of anti-bacterial and antibiotic agents from carriers containing soft vesicles has been considered as a safe and promising approach for the treatment of deep skin infections. This is owing to the ability of these carriers to carry the antibiotic into deep skin strata for eradication of bacterial infections. Godin and Touitou in a CLSM study showed that FITC-bacitracin administrated in ethosomes to rats, penetrated to the deep layers of the skin through the inter-corneocyte pathway in the SC. This efficient delivery was not observed when the molecule was applied in a hydroethanolic solution or in liposomes. These data emphasize the important role of the soft ethosomal vesicles in the delivery mechanism [14].

Touitou and Godin [15] investigated in vitro and in vivo an ethosomal erythromycin system to treat deep skin and soft tissue bacterial infections following topical drug application. In vitro susceptibility studies on the bacterial strains: *S. aureus* ATCC29213, *B. subtilis* ATCC6633, and *S. aureus*, showed that the ethosomal system yielded significantly larger inhibition zones and lower erythromycin minimum inhibition concentration (MIC) in comparison with hydroethanolic solution of the drug. Erythromycin ethosomal system applied topically to Swiss Albino ICR (CD-1) mice with *S. aureus* ATCC29213 infection, completely cured the infection. On the other hand, application of the antibiotic from a hydroethanolic solution was not efficient in inhibiting the infection. Histopathological examination indicated that animals treated with the hydroethanolic solution developed deep dermal and subcutaneous abscesses with necrotic destroyed skin and dense infiltrates of neutrophils and macrophages.

Bagchi et al. tested the antibacterial effect of ethosomes containing psoralen on cultures of *Escherichia coli*, *Staphylococcus aureus* and on bacterial biofilms. The cultures were exposed to UVA radiation for 30 min. As reported by the authors, application of psoralen from ethosomes reduced significantly the bacterial survival by ~70%. Destruction of the bacterial adherence in the biofilm was also observed. It was suggested the fluidizing effect of ethosome enhanced the permeation of into the bacterial cell. Such promising approach may open a new strategy for the treatment of multi-drug-resistant bacterial-induced skin diseases [39].

### 2.4. Treatment of Fungal Skin Infections

Fungal infections of the skin and nails account for a large percentage of the skin diseases. These infections vary in the severity from being superficial and potentially self-limited infections, such as the common dermatophyte infections, to severe conditions with dissemination or local invasion into deeper tissue. Many of the fungi affecting the skin can also cause nail infections called Onychomycosis. Topical antifungals creams of griseofulvin, terbinafine, fluconazole and itraconazole for several weeks are the most common treatment in the cases of fungal infections [82]

Ethosomal systems containing antifungal agents such as griseofulvin (GRF), clove oil and voriconazole were investigated. GRF, an antibiotic with activity against a wide spectrum of fungal infections, was incorporated in ethosomes by Marto et al. The ability of the carrier to deliver the compound deeply into/through newborn pig skin was investigated in vitro. The reported results indicated that almost 40% of the drug was found in the SC at the end of the experiment. These findings were confirmed by the fluorescence assay with Nile red-loaded ethosomes which evidenced its retention in this layer. The in vitro antifungal activity of GRF in ethosomes, tested against *T. rubrum* ATCC 28188, indicated marked fungicidal effect. On the other hand, empty ethosomes did not exhibit any antifungal activity [40].

In another study, the antifungal activity of clove oil ethosomes incorporated in a carbopol 974 gel was studied by Shetty et al. The ex vivo drug permeation into albino rat skin showed that a cumulative drug release of 83.11 ± 0.54% after 12 h application. The antifungal activity against *Candida albicans* measured in studies carried out using the cup plate method revealed a very significant (*p* < 0.01) antifungal activity for the ethosomal composition relative to pure clove oil. The authors suggest that the above described formulation could be promising in the topical delivery of clove oil for the treatment of fungal infections [41].

In a recent study, a modified ethosomal system containing the penetration enhancer, cinnamaldehyde, was investigated for dermal targeting delivery of terbinafine [83]. This system significantly improved the targeting efficiency leading to a drug deposition of around 18.5% in the deep skin layers compared to only 5.6% for a commercial Lamisil^®^ cream. The dermal targeting effect was further confirmed by visualization of rhodamine-labeled system across SC into the epidermis and the dermis layers of hairless rats. The skin irritation tendency, also evaluated in vivo on rabbits using the Draize scoring method, which tests erythema, edema and scar formation, indicated that sub-chronic application of the system for 7 days is safe and nonirritating. The antifungal activity of this system was proved in vitro on *Candida albicans* strains by minimal inhibitory concentration (MIC) assay. The reported results indicated that lower drug doses are required for efficient treatment of skin fungal infections as compared to DMSO solution of the drug. As suggested by the authors, this approach may improve the patient compliance and decrease the incidence and severity of side effects leading to better therapeutic outcome.

### 2.5. Treatment of Skin Inflammation

Skin inflammation is a major skin disorder requiring medical intervention. It is a complex process occurring in the body in answer to tissue damage. This condition can be provoked by pathogens, noxious mechanical and chemical agents and as an autoimmune response. Common symptoms of inflammation are redness, swelling, itching, heat, and pain [84]. Atopic dermatitis, or atopic eczema, is a common chronic inflammatory skin disease [85]. Treatment of atopic dermatitis comprises topical administration of glucocorticosteroids and immunosuppressants, such as tacrolimus and pimecrolimus. Microbial colonization and superinfection may induce disease exacerbation and can justify additional antimicrobial treatment [86]. Local treatment with these agents administrated to the skin from conventional carriers is not always effective.

Paolino et al. [45] evaluated the anti-inflammatory effect of the ethosomal system of ammonium glycyrrhizinate in a clinical study on twelve healthy volunteers. The anti-inflammatory activity and the tolerability of the system were evaluated using the non-invasive reflectance spectrophotometry method in healthy volunteers with methyl nicotinate induced skin erythema. An ethosomal formulation containing 0.3% *w/v* ammonium glycyrrhizinate was administrated to the participants affected area and compared to the administration of hydroethanolic and aqueous solutions of the active molecule. The measured erythema index (ΔEI) was 29.6% for the ethosomal system versus 62.7 and 60.7% for the comparative ethanolic and aqueous solutions, respectively.

In another work, ethosomal compositions of matrine, triptolide, apigenin and crocin were designed and tested in vivo on animal models of skin inflammation. The effect of the carrier on the percutaneous permeation of the compound in vitro and its anti-inflammatory activity in vivo in rat skin were studied. A rapid and effective anti-inflammatory effect of the tested active molecules incorporated in the ethosomal carrier were reported [46,47,48,87].

Recently, Andleeb et al. [53] incorporated in ethosomes containing ethanol and propylene glycol and in an ethosomal gel the antioxidant extract of *Achillea millefolium* L. In vitro drug release study using Franz diffusion cells and fresh rat skin indicated cumulative peremeated compound amounts of 78.6, 79.8, 30 and 28.7% from the ethosomal formulation, the ethosomal gel, a hydroethanolic extract and a conventional gel, respectivley.

### 2.6. Treatment of Psoriasis

Psoriasis is a chronic, inflammatory, hyperproliferative immunologically mediated skin disease with a genetic origin. Epidermal hyperplasia and altered keratinocyte differentiation are the prominent features of this disorders. In most of the patients, these clinical presentations turn into chronic plaque psoriasis or develop to nail disease, and/or an arthritis, causing pain and skin deformation. At the microscopic scale, psoriasis is characterized by increased proliferation and incomplete differentiation of the epidermal skin layer, a marked increase in cutaneous blood flow, and leukocytic infiltration in the papillary dermis and in the epidermis [88].

Psoralen ethosomal systems were investigated for psoriasis therapy. In an in vitro skin permeation study carried out in Franz diffusion cells and excised rat skin, the drug delivery rate from the ethosomal system versus liposomes was evaluated. The results indicated a transdermal flux and a skin deposition of 38.89 mg/cm^2^/h and 3.87 mg/cm^2^, respectively, for the ethosomal system. These values were 3.5 and 2.1-folds higher than those achieved for liposomes containing an equal drug concertation. A better biocompatibility with human embryonic skin fibroblasts was reported for the ethosomal system having a viable cell count of 61% (*p* < 0.05). The authors suggest that ethosome could be considered as potential carriers for psoralen and other anti-psoriatic agents [54].

An ethosomal gel containing methotrexate and salicylic acid was investigated for the treatment of imiquimod-induced psoriasis in mice. The treatment efficacy was evaluated by scoring the Psoriasis Area and Severity Index (PASI) and histopathological examination. The results of the in vivo experiment indicated that following 7 days administration of the two drugs in ethosomal system, a maximum PASI was achieved with less erythema and skin thickening as compared to untreated or Vaseline treated animals. The histopathologic report indicated a normal skin with mild keratosis in the group treated with the ethosomal gel, whereas signs of moderate to high hyperkeratosis as well as orthokeratosis were observed in the group treated with a blank gel [55].

In a recent study by Fathalla et al. [56], ethosomal and liposomal systems of the anti-psoriatic agent, anthralin, were prepared aiming to enhance the efficacy the drug and reduce its notorious side effects. The clinical efficacy and safety of the systems incorporated in a Pluronic^®^F-127 gel was evaluated in 20 psoriasis patients (16 males and 4 females). The results indicated that at baseline, the patients in the ethosome group had a median Psoriasis Area and Severity Index (PASI) of 3.6 as compared to 3.4 for liposome. Upon treatment, the PASI scores for patients treated with ethosomes and liposomes were 81.84 and 68.66%, respectively (*p* < 0.05). No side effects were reported for the two treatments. These finding show that anthralin ethosomes could be considered as a potential safe and effective treatment for psoriasis.

A combined therapy of curcumin ethosomes modified with glycyrrhetinic acid-D-α-tocopherol acid polyethylene glycol succinate was recently investigated for topical treatment of psoriasis by Gou et al. [57]. Using an interleukin-6-induced cell model of oxidative stress, the authors found that the new system displayed a desirable anti-inflammatory effect by downregulating the expression of oxidative stress proteins. After topical administration to imiquimod induced psoriatic mice, the combined system reversed the progression of psoriasis-like skin lesions and accelerated the process of skin healing as compared to controls lacking this combination. It was suggested that this combination approach generated a synergetic effect of curcumin ethosomes and glycyrrhetinic acid.

### 2.7. Treatment of Skin Cancer

Skin cancer accounts for most malignancies across the world. Melanoma and non-melanoma (basal and squamous cell carcinoma) skin cancer are the most common types of cancer affecting the skin tissue. Fair skin and chronic ultraviolet B exposure are believed to be the most important risk factors [89,90].

Squamous cell skin cancer may be preceded by premalignant lesions called actinic keratosis. In such cases, chemical destruction of the superficial lesions by topical application of the pyrimidine antagonist, 5-fluorouracil (5-FU) is carried out. This agent is available in several topical conventional formulations, including 1, 2 and 5 percent solutions and 1 and 5% creams.

In cases of basal cell carcinoma, cryotherapy, curettage with electrodesiccation or radiation therapy are the most applied treatments. Other therapies include oral retinoids, topical tretinoin and topical 5-FU [91].

**Figure 6:**
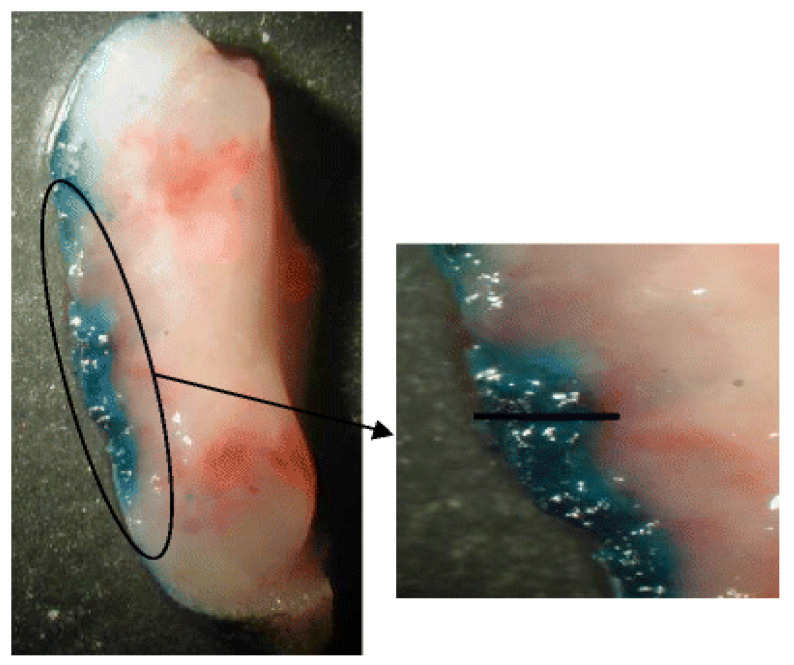
Delivery of methylene blue into the tumor from Tumorep. Photomicrographs showing delivery into the tumor of methylene blue following 4 h of application of Tumorep gel containing 1% methylene blue (bar = 1 mm). Efficient delivery of the colored probe into the tumor after 4 h is shown (Reprinted with permission from ref. [16]. Copyright 2011 Springer).

In an in vivo study, 5-FU was incorporated in a transethosomal carrier containing n-decyl methyl sulfoxide, called Tumorep, designed for efficient anticancer activity. In this investigation, Tumorep was tested on five cell types: three tumorigenic (TE.354.T, ES-2, and Mel624), one precancerous (HaCaT), and a primary keratinocyte (human normal keratinocytes) cell culture. A significant decrease in the viability of the tumor cell lines was reported. In addition, keratinocytes treated with the system exhibited a decreased percentage of keratin 14-positive cells, suggesting system ability to induce cell differentiation. Furthermore, the composition was tested in vivo on a mice model of skin tumor. Tumorep was applied daily and compared to Efudex, a commercial product containing 5% of the drug and to a control (untreated) mice group. The obtained results indicated that, the tumor volume was reduced to 285 ± 23 mm^3^ in mice treated with 5-FU Tumorep compared to 390 ± 22 and 1153 ± 273 mm^3^ in the animals treated with the commercial product and untreated animals, respectively. To understand this improved antitumor effect of the drug when applied in the new system, the delivery of methylene blue (having a blue color) from Tumorep into the tumor tissue was investigated. Cryo-sections of basal cell carcinoma skin tumor area treated with the Tumorep gel containing 1% methylene blue were examined. Figure 6 shows an intense blue color penetration into the tumor area 4 h after the system application. These results suggest that the ethosomal system could enable efficient penetration of the drug deeply into the tumor area leading to an efficient treatment [16].

Paolino et al. [58] tested the in vitro effect of paclitaxel ethosomes on human squamous-cell-carcinoma line (DJM-1). Entrapped paclitaxel at a drug concentration of 0.5 μM led to cellular mortality of ~40% of the cultures, after 48 h. This value is more than 2-folds greater than that obtained when drug was applied in the physical mixture at a same dose. The in vitro percutaneous permeation profile, tested in human skin using Franz diffusion cells for 24 h, revealed a dermal paclitaxel accumulation of 103.5 µg/cm^2^ for ethosomes loaded with the drug versus 20.35 and 4.31 µg/cm^2^ for physical mixture of the drug with the carrier, and hydroethanolic solution, respectively. These results indicate that paclitaxel entrapped in ethosomes possesses efficient anti-proliferative activity in the tested carcinoma cell line and an improved drug skin delivery.

The use of glycerosomes for the treatment of skin cancer was investigated in a recent study by Md et al. [60]. Plumbagin, a phytochemical with cytotoxic properties, was incorporated in glycerosomes aiming to overcome the poor solubility of the compound and to improve its low bioavailability. Optimized plumbagin glycerosomes were incorporated in a Carbopol 940 gel and their permeability enhancement property was tested ex vivo on rat skin using Franz diffusion cell. In a CLSM study using rhodamine B, a significant accumulation of the probe was found at a skin depth of 173.56 µm. The maximum depth of penetration of this probe delivered from a solution was only 12.45 µm. In addition, the IC50 values for the drug, as measured by MTT assay on murine tumor cell line (B16-F10), were 4.1, 10.4 and 19.1 for the glycerosomal system, conventional liposomal system and for a drug suspension, respectively. These data indicate a higher cytotoxicity of the glyecerosomal system on the tested tumor cells.

### 2.8. Treatment of Melasma and Vitiligo

Melasma is a hyper-pigmentation disorder of the face involving the cheeks, forehead and commonly the upper lip. This condition is more common in women which account for 90% of all cases. Topical hypo-pigmentating agents comprise hydroquinone (HQ), tretinoin (RA), kojic acid, and azelaic acid. Physical therapies, such as chemical peels (glycolic acid [GA], trichloroacetic acid [TCA]) are common treatments of this condition [92].

Celia et al. aimed to increase the dermal delivery of linoleic acid using ethosomes for improved management of melasma. For this purpose, the in vitro percutaneous permeation through human skin of the compound, loaded in the two vesicular systems, was evaluated as compared to hydroethanolic solution. The results show that the amount of linoleic acid permeated at the end of the 24 h experiment exceeded the 80% when ethosomes were used. The percutaneous permeation of linoleic acid was 237.75 and 195.15 μg/cm^2^ from the ethosomal formulations containing 40 and 45% ethanol, respectively. On the other hand, a value of 39.61 μg/cm^2^ was obtained for the hydroethanolic solution of the same molecule concentration. These results show the ability of ethosomes to significantly (*p* < 0.05) improve the percutaneous permeation of linoleic acid. The findings of this investigation suggest the suitability of ethosomes as drug carriers for topical treatment of skin hyperpigmentation disorders [64].

Vitiligo is a depigmenting disorder that is characterized by patchy or nonsegmental depigmentation. Topical therapies, including corticosteroids and immunosuppressants, may be effective for the treatment of localized vitiligo [93]. Methoxsalen is a medication used for management of vitiligo due to its ability to induce melanin production in the skin upon exposure to ultraviolet light. Garg et al., [65] designed a methoxsalen ethosomal gel. Results of the ex vivo study on rat skin, indicated that the system was able to improve the accumulation of methoxsalen in the dermal and epidermal layers. The measured skin deposition of the compound in the deeper dermis layer was 21.64 ± 0.11 mg·cm^−2^ when administrated in ethosomes, compared to half of the amount for the control formulations. Further, in in vivo skin permeation work with 123 rhodamine applied in ethosomes was delivered to the deep skin layers as examined by fluorescence microscopy. This enhanced delivery of the probe was not observed for the compound administrated in a hydroethanolic solution. Furthermore, methoxsalen ethosomal system reduced remarkably the phototoxic effect of UV light on rat skin in vivo.

### 2.9. Wound Healing

Wound healing is a complex and highly regulated process that is critical for maintaining the barrier function of skin. Although the mechanisms underlying wound healing are not fully understood, the anti-inflammatory and anti-microbial mediators are believed to play a key role in this condition [94].

In an in vivo study by Partoazaret al. [61], the effect of 2-weeks treatment with curcumin ethosomal system on the wound healing and bacterial flora in rats with second degree skin burn was evaluated. The curcumin ethosomal treatment led to a significant recovery of the wound and re-epithelization (*p* < 0.01), neovascularization (*p* < 0.01), collagen synthesis (*p* < 0.001), granulation tissue formation (*p* < 0.001) as compared with untreated control animals. The ethosomal formulation of curcumin and the silver sulfadiazine cream both inhibited (*p* < 0.001) the growth of the burn bacterial flora. In addition, the antibacterial activity of the ethosomal system was estimated to be approximately 11% higher than that of the free curcumin as shown by the reduction of the burn bacterial flora.

In another work curcumin-propylene glycol liposomes were evaluated for the treatment of animals subjected to second degree burns. Wound repair was evaluated histopathologically after eight days of treatment, according to Abramov’s scoring system. The authors found at the end of the application time of curcumin system on burned rats, a significant wound repair was observed (*p* < 0.001) as compared to the application of conventional liposome and free curcumin. The epithelization, angiogenesis and fibrosis scores were more than 1.5 folds higher for the modified liposome group as compared to the control groups. The inflammation score was the lowest for the ethosomal system, with a score of 1, as compared to more than 2 for the free drug and for the rigid liposome. In addition, the antibacterial activity of the propylene glycol liposomal formulation was similar to that of silver sulfadiazine cream 1% [62].

Fu et al. investigated an ethosomal system of thymosin β-4 (Tβ-4), a macromolecule for wound repair, in vivo on a mice model of skin injury [63]. Optimized Tβ-4 ethosomal gel shortened the wound healing time by half as compared to that of a T-β4 gel. Compared with the free drug animal group, the ethosome preparation promoted the percutaneous absorption of the macromolecular protein and shortened the wound recovery time.

### 2.10. Treatment of Hair Loss

Hair loss is a common and stressing condition that often leads to decreased self-confidence and in some cases may cause personality and psychological problems. The most common type of hair loss is androgenic alopecia, a hereditary androgen induced hair loss condition. This hair loss problem is usually treated with Finasteride or Minoxidil in men and women in addition to estrogen and Spironolactone in women. Alopecia areata is an autoimmune disease affecting the hair growth. Glucocorticoids, minoxidil, anthralin, plant extracts and topical immunosuppressants are the treatment choices in such cases [95].

Enhanced delivery of the above-mentioned molecules to target pilosebaceous units is one of the most promising strategies for the treatment of hair loss [66]. Drug ethosomal systems have been investigated in this field.

The first drug impeding hair loss incorporated in carriers containing soft vesicles was minoxidil [10]. In vitro drug application of minoxidil ethosome to excised nude mice skin resulted in a drug permeation of 637.0 ± 92.0 µg/cm^2^. This amount is 10, 45 and 35 times higher compared to the permeation from a 2% phospholipid ethanolic solution, a hydroethanolic solution or from absolute ethanol, respectively. The skin localization of minoxidil was two, seven and five-folds higher from ethosomes as compared to the above-mentioned control systems, respectively. In addition, the application of minoxidil ethosomes to hairless mouse skin in vitro led to accumulation of the drug in the hair follicles and in the sebaceous glands as analyzed by quantitative autoradiography [66]. These results suggest that using the ethosomal carrier, the hypertrichotic effect of minoxidil may be improved.

In another study in this field, ethosomal systems loaded with the leaves extract of *Phyllanthus niruri*, the rhizomes of *Zingiber ofcianale*, and *Croton tiglium linné* seeds were evaluated for their hair growth promoting activity. In vivo studies were performed on male rats with testosterone-induced alopecia and compared to the effect of finasteride. The animals received concomitant treatment with ethosomal systems of the extracts or finasteride and a testosterone subcutaneous injection for 21 days. The results indicated that the extract of C. tiglium loaded ethosomal formulation and ethanolic extract of *P. niruri, Z. officianale, C. tiglium* loaded ethosomal formulation yielded hair follicle densities of 2.75 ± 0.75 and 2.41 ± 0.90 no./mm, respectively. The values achieved by the commercial treatment (Finasteride) were 3.3 ± 0.77 no./mm. Animals in the group of testosterone negative control group exhibited a hair follicle density of only 1 ± 0.4 no./mm [68].

### 2.11. Treatment of Skin Aging

Human skin aging is caused by a number of factors. Exposure to UV radiation is one important condition and leads to oxidative stress and damage of the skin’s structure and integrity. In addition, it can cause visible skin aging including dryness, loss of elasticity, wrinkles, discoloration, and changes in texture, the effect of sunlight on the skin is an increased incidence of precancerous conditions and skin cancer [96].

Vitamin E is an antioxidant molecule with photoprotective characteristics. An interesting approach to enhance the photoprotective effect against UV radiation of this vitamin is applying it on the skin incorporated in ethosomal formulations. The authors suggested that it would be beneficial to target the delivery of the incorporated vitamin into the intracellular compartments of deep dermal layers, where the photoprotective effect is needed. Efficient intracellular delivery and dermal accumulation of Vitamin E were achieved by administration of ethosomal systems. The system led to an important accumulation of the molecule within the skin (up to 54% of the applied dose). The permeation of vitamin E to the dermal fibroblasts was 3.5 times higher from ethosomes than from liposomes [69]. Further, an interesting clinical strategy for prevention of skin photoaging is suggested by the authors, in which nonpermeating sunscreens are used during the direct UV exposure and followed by the application of the ethosomal system of vitamin E.

Jeswani and Saraf investigated the anti-wrinkle effect of ethosomes containing curcumin system. The results of this work indicated that multiple application of system for two weeks to healthy human volunteers resulted in 10 to 50% improvement in viscoelasticity, total deformation, biological elasticity and sagginess of the skin [70]. In another work, the ethosomal system of the protective antioxidant agent, coenzyme Q10, was evaluated in fibroblasts and 3D reconstituted human epidermis with hydrogen peroxide induced oxidative stress. The results showed that this pretreatment counteracted the oxidative stress and prevented the formation of the oxidative damage biomarker 4-hydroxynonenal protein [97].

Yücel et al. [71] prepared and investigated ethosomes containing rosmarinic acid. This active ingredient is a natural compound used as a free radical scavenger to prevent the formation of lipid peroxides, which is caused by UV rays and other physical/chemical external factors. The antioxidant activity, measured at the end an ex vivo permeation experiment using Franz diffusion cells through full thickness mouse skin, indicated an inhibition of collagenase activity of 71.7% for the ethosomal group as compared to 67.6% for the skin treated with rosmarinic acid from liposome. In addition, the inhibition of elastase enzyme activity was reported to be 79.1 and 76.2% for ethosome and liposomes, respectively. The authors suggested that ethosomes act more effectively than liposomes against free radicals-related skin aging.

## 3. Products Based on Carriers Containing Phospholipid Soft Vesicles

Products designed with these carriers include Supravir (Trima, Kibbutz Ma’abaro, Israel) an ethosomal cream of acyclovir for the treatment of herpes simplex and a number of ethosome based products for reduction of skin cellulite such as Noicellex (Novel Therapeutic Technologies, Herzliya, Israel), Skin Genuity, (Physonics, Nottingham, UK), Lipoduction (Osmotics, New York, NY, USA) and Cellulight EF (Hampden Health, Wahroonga, Australia). Decorin Cream (Genome Cosmetics, Bensalem, PA, USA) an anti-aging and skin repair cream and Nanominox (Sinere, Ludwigshafen, Germany) a hair growth promoter, were also developed based on ethosomal compositions.

## 4. Conclusions

We reviewed here work carried out with systems containing phospholipid soft vesicles for dermal treatment of skin medical conditions. Drug incorporation in ethosomes led to improved management of acne vulgaris, skin infections, skin inflammation, psoriasis, skin cancer, hair loss, skin injury and skin aging, as shown in numerous in vitro, in vivo and in clinical studies. Transethosomes and glycerosomes, had the ability to enhance the in vitro penetration of the incorporated active molecules.

Given the large amount of published studies including several clinical trials, we expect that more products will be launched on the market.

This review will guide and help researchers, dermatologists, pharmacists, formulators and students to select a suitable carrier for their drug in order to achieve an efficient dermal treatment of a specific skin disorder.

## Figures and Tables

**Table 1:** Skin disorders treated with active molecules incorporated in carriers containing phospholipid soft vesicles.

Treated Disorder	Investigated Vesicular Carrier	Study	Ref.
Acne Vulgaris	Clindamycin and salicylic acid ethosomal system	Clinical study on the reduction of acne vulgaris and skin tolerability of the formulation	[31]
	Azelaic acid ethosomal system	Diffusion study through synthetic membrane	[32]
	Cryptotanshinone ethosomal system	In vitro skin permeation and skin deposition; in vivo anti-acne activity on rabbits	[33]
	Karanjin ethosomal system	In vitro skin premeation study on excised rats skin; in vivo skin irritation study on rats; in vivo anti-inflammatory and anti- acne studies on rats	[34]
Viral infections	Acyclovir ethosomal system	Two-armed double-blind clinical study on subjects with recurrent herpes labialis	[35]
	Acyclovir ethosomal system	Antiviral activity against HSV-1 by plaque reduction assay in monolayer cultures of Vero cells	[36,37]
	9-[(2-hydroxyethoxy) methyl]guanine ethosomal system	Antiviral activity against HSV-1 by plaque reduction assay in monolayer cultures of Vero cells	[37]
	Ethosomal system of the essential oil of *Melissa officinalis* L.	In vitro activity against HSV type 1 in mammalian cells	[38]
Bacterial infections	Bacitracin ethosomal system	Intracellular penetration and localization in fibroblasts (3T3); in vitro deposition and permeation through human cadaver skin	[14]
	Erythromycin ethosomal system	In vivo activity in mice model of deep dermal *S. aureus* infection	[15]
	Psoralen ethosomal system	Photodynamic therapy in biofilms formed in Petri dishes	[39]
Fungal infections	Voriconazole transethosomal system	In vitro skin permeation and deposition studies through mice skin; in vivo deposition study on mice	[12]
	Griseofulvin ethosomal system	In vitro permeation and deposition study on new-born pig skin	[40]
	Clove oil ethosomal system	Ex vivo permeation studies on rat skin; antifungal activity in cup plate test against *Candida albicans*	[41]
	Voriconazole ethosomal system	In vitro anti-fungal activity against *Asperigillus flavus* colonies. In vitro skin deposition and permeation through abdominal rat skin	[42]
	Econazole nitrate transethosomal system	Ex-vivo skin permeation and retention studies followed by in vitro antifungal activity *against C. albicans* fungus	[43]
Skin inflammation	Diclofenac ethosomal system	In vitro permeation study on rat skin; in vivo anti-inflammatory activity in carrageenan-induced rat paw edema model	[11,44]
	Ammonium glycyrrhizinate ethosomal system	In vitro permeation through human skin; clinical study to evaluate the anti-inflammatory activity in volunteers with methyl nicotinate erythema	[45]
	Matrine ethosomal system	In vitro percutaneous permeation study on rat skin; in vivo anti-inflammatory activity in rat measured by reflection spectrophotometery	[46]
	Apigenin ethosomal system	In vitro and in vivo deposition study on rat skin; evaluation of the reduction of cyclooxygenase-2 levels in mouse with skin inflammation	[47]
	Crocin ethosomal system	Evaluation of the anti-inflammatory activity on healthy volunteers.	[48]
	Diclofenac ethosomal system and Diclofenac transethosomal system	In vitro permeation and deposition studies on rat skin	[49]
	Diclofenac glycerosoomal system	In vitro penetration and permeation studies on new-born pig skin	[13,50,51]
	Paeoniflorin glycerosomal system	In vitro permeation experiments through excised rat abdominal skin; in vivo deposition in rat synovium	[52]
	*Achillea millefolium* L. extract ethosomal system	In vitro permeation strudy through fresh rat skin	[53]
Psoriasis	Psoralen ethosomal system	In vitro permeation and penetration study using Franz diffusion cells and excised rat skin	[54]
	Methotrexate and Salicylic acid ethosomal system	In vitro retention and permeation study on pig ear skin; in vivo anti-psoriatic activity in mice model with imiquimod-induced psoriasis	[55]
	Anthralin ethosomal system	Preparation, comparative evaluation and clinical assessment in psoriatic patients	[56]
	Curcumin ethosomal system surface-modified with glycyrrhetinic acid-D-α-tocopherol acid polyethylene glycol succinate	In vitro anti-inflmmatory effect on interleukin-6-induced oxidative stress cell model; in vivo anti-psoriatic activity in mice model with imiquimod-induced psoriasis	[57]
Skin cancer	5- Fluorouracil ethosomal system containing -decyl methyl sulfoxide (Tumorep)	In vitro anti-tumor effect on five cell lines; in vivo anti-tumor effect in mice model of skin cancer	[16]
	Paclitaxel ethosomal system	In vitro permeation study on human SC; in vitro antiproliferative effect in squamous carcinoma cells	[58]
	Fe-chlorophyllin transethosomal system	In vitro skin permeation and deposition studies through mice skin; in vivo evaluation of the anti-cancer effect in mice	[59]
	Plumbagin glycerosomal system	Ex vivo permeation study on rats skin	[60]
Skin injury (wound healing)	Curcumin ethosomal system	In vivo wound healing effect in rats	[61]
	Curcumin-propylene glycol liposomal system	In vivo wound repair effect is rats with burned skin	[62]
	Thymosinβ-4(Tβ-4) ethosomal system	In vitro drug release study on mice skin; in vivo pharmacokinetic and skin irritation studies on mice	[63]
Skin pigmentation disorders	Linoleic acid ethosomal system	In vitro percutaneous permeation through human stratum corneum and viable epidermis membrane	[64]
	Methoxsalen ethosomal system	Ex vivo release studies and photo- toxicity after exposure to UV light	[65]
Hair loss	Minoxidil ethosomal system Minoxidil glycerosomal system	In vitro penetration and permeation through abdominal nude mice skin	[10,66,67]
	Ethosomal systems of plant extracts	In vivo effect on hair growth in rats with testosterone induced alopecia	[68]
Skin aging	Vitamin E ethosomal system	In vitro permeation studies through skin and cultured fibroblasts	[69]
	Curcumin ethosomal system	Clinical trial evaluating skin viscoelasticity, total deformation, biological elasticity and sagginess	[70]
	Rosmarinic acid ethosomal system	Ex vivo permeation studies using Franz diffusion cells and mice skin; ex vivo antioxidant activity	[71]

## Data Availability

Not applicable.
